# Sexual behavior and perceived risk of HIV AIDS among returnee labor migrants from Overseas in Nepal

**DOI:** 10.1186/1742-4690-9-S1-P106

**Published:** 2012-05-25

**Authors:** Sushma Dahal, Paras Kumar Pokharel, Birendra Kumar Yadava

**Affiliations:** 1 School of Public Health and Community Medicine, BPKIHS, Dharan, Nepal

## Introduction

Nepal is a popular country of origin for labour migrants. Migrant workers are risk group for HIV. In Nepal studies on labour migrants have mainly focused on those going to India. Not enough studies have focused on sexual behaviour of migrants going to overseas.

## Materials and methods

A cross sectional study was done among 110 returnee male labour migrants in Nepal who were interviewed about their sexual behaviour while in overseas and their perceived risk of HIV/AIDS. Recruitment agencies were selected purposively to identify returnee migrants. Snowball technique was also used to trace some migrants in houses and hotels.

## Results

Saudi Arabia, Malaysia, UAE and Qatar were found to be the popular countries of destination. Among respondents returning from Gulf countries, 41.6% had sex with paid/unpaid partner which was 66.7% for those from Non-Gulf countries. Among 21 respondents who didn’t always use condom, 15 were from Gulf countries and 6 were from Non-Gulf countries. Respondents for whom difficulty in finding condom was the reason for non use were from Gulf countries. Co-working female friends were the non spousal unpaid partners for majority of the respondents. There was not much difference in risky sexual behaviour of migrants based on law of destination country regarding sex work and respondent’s habit of drinking alcohol. AIDS was perceived to be a very dangerous killer disease by more than 2/3rd of the respondents; even though, more than 40% perceived themselves to be at some risk and more than 75% perceived their friends to be at some risk of HIV. Only 7% had ever heard and used VCT service.

## Conclusions

Labour migrants going to overseas are at risk of HIV. HIV related awareness raising activities should focus on migrants. HIV prevention programs in destination and origin country should target both male and female labour migrants.

